# Case series of recurrent inguinal hernia after primary TREPP repair: *re*-TREPP seems feasible and safe

**DOI:** 10.1016/j.ijscr.2018.08.060

**Published:** 2018-09-12

**Authors:** A.M. Persoon, W.J.V. Bökkerink, W.L. Akkersdijk, C.J.H.M. van Laarhoven, G.G. Koning

**Affiliations:** a Dept of Surgery, St. Jansdal Hospital, Wethouder Jansenlaan 90, 3844 DG Harderwijk, the Netherlands; b Dept of Surgery, Radboud UMC, Geert Grootteplein Zuid 10, 6525 GA Nijmegen, the Netherlands; c Dept of Surgery, Noordwest Hospital Group, Wilhelminalaan 12, 1815 JD Alkmaar, the Netherlands

**Keywords:** Inguinal hernia, Recurrence, TREPP, Chronic postoperative pain

## Abstract

**Introduction:**

The Trans REctussheath PrePeritoneal (TREPP) mesh repair was introduced in 2006 to decrease the risk of postoperative inguinal pain in hernia surgery. For the repair of a *recurrent* inguinal hernia after a primary TREPP an alternative open anterior route (Lichtenstein) may seem the most logical option, but coincides with an increased risk of chronic postoperative inguinal pain. Therefore, this study aimed to evaluate the feasibility of a *second* TREPP procedure to repair a recurrent inguinal hernia after an initial TREPP repair.

**Methods:**

Consecutive patients with a recurrent inguinal hernia after a primary TREPP, repaired by a *re-*TREPP were retrospectively included in the study. Data, retrieved from the electronic patient files, were combined with the clinical findings at the outpatient department where the patients were physically investigated according to a priorly written and registered protocol.

**Results:**

Overall about 1800 TREPPs were performed between 2006 and 2013. Since the introduction of TREPP in 2006, 40 patients presented with a recurrence in our center. From this group 19 patients were *re-*operated with the TREPP technique. No intraoperative complications occurred. There was one conversion from *re-*TREPP to Lichtenstein and no *re-*recurrences occurred to date. Ten out of 19 patients could be clinically evaluated with a mean follow-up period of 37 months (range 11–95). None of these patients (*n=*10) complained of chronic postoperative inguinal pain. Two patients reported discomfort. One patient died non procedure related, three weeks after re-TREPP of sudden cardiac death.

**Conclusion:**

These first experiences with re-TREPP for secondary inguinal hernia repair are encouraging for the aspects of feasibility and safety, particularly in experienced surgical hands.

## Highlights

• Recurrent inguinal hernias are preferably treated via an alternative route, e.g. posterior after anterior.
• Endoscopic preperitoneal repair techniques are common for groin hernias after Lichtenstein’s plasty.
• The TREPP technique is a minimal access, open variant of these preperitoneal techniques.
• The TREPP technique seems to be a safe and feasible technique to use for recurrence after previous TREPP for inguinal hernia repair.

## 1 Introduction

Inguinal hernia repair is frequently performed with approximately 30,000 repairs each year in the Netherlands and 730,000 cases in the USA annually [1,2]. The risk of recurrence depends on many factors and ranges in literature from 0 to 7.7% after at least two years of follow-up [3,4], Level of Evidence (LoE) 2a [5]. Chronic Postoperative Inguinal Pain (CPIP) occurs frequently when using the Lichtenstein technique, estimated risk 11% (range 0–43% (LoE 2a)) [6]. The Trans REctussheath PrePeritoneal mesh repair (TREPP) was developed in 2006 as an open preperitoneal technique to reduce CPIP [[7], [8], [9]]. To ensure the safety and efficacy critical evaluation is needed [10,11]. According to the recommendation in the latest international guideline an alternative operation route is preferred to avoid complications due to distorted anatomy [3]. Therefore an open anterior onlay mesh technique such as Lichtenstein’s can be considered as a reliable technique for secondary inguinal hernia repair (SIHR) after a previous preperitoneal approach such as TREPP. The risk of CPIP is higher in recurrent hernias [12] giving an extra reason to avoid contact with the inguinal nerves [13]. Experienced inguinal hernia surgeons performed *repeated* TREPP (*re*-TREPP) in a group of patients with a recurrence after a primary TREPP mesh repair. TREPP is educated and many surgeons in training for TREPP ask if re-TREPP is possible. Therefore we investigated this demanding and repetitive question. Aim of this study was to retrospectively investigate the feasibility of a *re-*TREPP evaluating the incidence of patients with CPIP, the rate of *re-*recurrence and other complications.

## 2 Methods

Since the introduction of TREPP in 2006, TREPP has been the standard operation technique for all patients who present in our center with an inguinal hernia. All consecutive patients who were operated at our non teaching hospital in the Netherlands, via TREPP between January 2006 and December 2013 were investigated. Information was taken from the notes of telephone consultation 2 and 30 days postoperatively, which is the local standard protocol for follow-up. Adult patients who presented themselves to this hospital with a recurrent inguinal hernia after TREPP were selected. Within this group, patients who had undergone a *re-*TREPP were subsequently included in this study. These *re-*TREPP patients were invited for a follow-up visit at the outpatient department with physical examination, or they were visited at home by the investigator. The minimal postoperative period for follow-up was set at one year after the secondary intervention (*re-*TREPP). Written informed consent was obtained from all patients. Recurrent inguinal hernia was defined as a reappearance of the inguinal hernia, diagnosed by physical examination (a reducible bulge with positive Valsalva).

### 2.1 Surgical technique

The *re-*TREPP procedures were all performed by two dedicated expert hernia surgeons. The principle of the *re-*TREPP for SIHR was comparable to a TREPP for primary inguinal hernia repair (PIHR), as described [7,8].

The standard operation procedure for TREPP and re-TREPP are well known among the surgeons who operate. In short: After opening PPS, the first mesh was identified and aspects of wrinkling, folding or malposition were assessed. The hernia was usually reduced by the development of the PPS by lifting the peritoneal sac with two long retractors, in line with the TREPP for PIHR. An additional mesh with memory ring was inserted (PolySoft^®^ hernia patch ‘Large’, Bard, IJsselstein, the Netherlands). Removal of the old mesh was avoided when possible to minimize the risk of nerve and vessel damage in the groin region. The mesh was only spared, as long as reposition was not hindered and the second mesh could be inserted in a correct, flat, PPS position. If the second mesh seemed to migrate easily or had limited overlap to the previously placed mesh, one or two standard absorbable stitches were used to fixate the mesh to the old mesh.

Baseline data such as state of the mesh during operation (meshoma, wrinkling of the mesh, malposition), type of hernia (medial or lateral) and number of conversions to other techniques were extracted from the electronic patient files and the operation reports. Clinical parameters such as *re-*recurrence rates, incidence of CPIP, sexual complaints (presence of peri-ejaculatory pain) and sensory disturbances were clinically evaluated and/or asked. Sensory disturbances were measured with the pin-prick test and drawn on a dermatome map [14]. Disturbances of more than 0.5 cm from the scar were considered relevant. Patients were asked to categorize any complaints as either ‘discomfort’ or ‘pain’. Discomfort was defined as any unpleasant but non-painful sensation that “irritated” or “annoyed” the patient. In case of pain, the Visual Analogue Scale (VAS) was used to measure pain intensity in rest and during activity, and the Pain Disability Index (PDI) [15] was used to measure interference of inguinal pain in daily life. All patients who reported pain related to the surgery were classified as chronic pain patients, regardless of the VAS-score. Sexual dysfunction was addressed whilst taking the patients history in a standardized way. This study was conducted and reported in line with the Preferred reporting of case series in surgery guidelines (PROCESS) [16]. Written informed consent was obtained from the patients for publication of this case series and accompanying tables. A copy of the written consent is available for review by the Editor-in-Chief of this journal on request.

## 3 Results

**Fig. 1:**
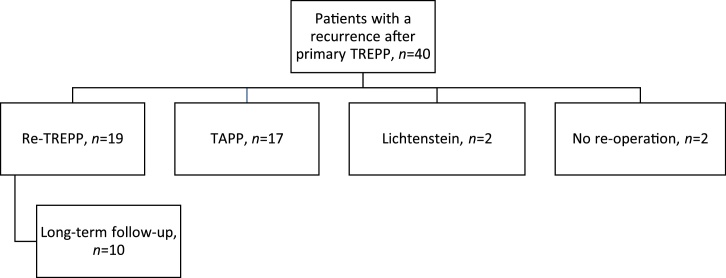
Flowchart of type of operation performed in patients presenting with a recurrence after TREPP.

Between January 2006 and December 2013 approximately 1800 patients were operated via the TREPP technique. A total of 40 patients were diagnosed with a recurrent inguinal hernia after an initial TREPP repair (Fig. 1). With this incidence it was assumed to be inappropriate to calculate the recurrence rate since patients might have went to another hospital with a recurrence. Nineteen of the forty patients with a recurrent hernia were operated with a *re-*TREPP procedure as described.

From all *re-*TREPP patients (*n=*19), the mean age at time of the *re-*operation was 68 years (range 56–86 years). All included patients, 18 males and 1 female, were classified with the American Society of Anesthesiologists (ASA) classification ≤3. The mean operation time was 41 min (range 17–96 min). All could be treated in day care. The mean time between first and second TREPP operation was 546 days (range: 12–2373 days). In five patients the *re-*TREPP was performed within 30 days because of malposition of the mesh (*n* = 3), folding (*n* = 1) or reasons not otherwise specified (*n* = 1). Most recurrences were lateral (*n=*10), seven recurrences were medial hernias and two operation reports did not mention classification for unknown reason. The status of the mesh in the PPS was established during *re-*TREPP repair (Table 1). The ‘old’ mesh was removed in one patient in whom reposition of the hernia sac was impossible until the mesh was removed. In ten patients the new mesh was fixated to the old mesh with a non-absorbable Prolene suture (Prolene®, Ethicon, part of Johnson and Johnson, New Jersey, U.S.A.) or to a surrounding tissue structure with a absorbable Vicryl suture (Vicryl®, Ethicon, part of Johnson and Johnson, New Jersey, U.S.A.). Almost all patients were operated under spinal anaesthesia (*n=*16). Table 2 shows the short- and long-term outcomes after *re-*TREPP. One *re-*TREPP was converted into a Lichtenstein’s, due to severely distorted anatomy. One patient with known hypertension and angina pectoris died from sudden cardiac death 3 weeks after his *re-*TREPP. No *re-*recurrences occurred in the first 30 days postoperatively. No *re-*operations were required. One patient was treated for a superficial wound infection with antibiotics and two patients developed an haematoma or seroma.

**Table 1:** Status of the mesh during re-TREPP repair.

	Total number of patients
Meshoma or folding	6
Malposition	9
Not otherwise specified	4

**Table 2:** Adverse events of re-TREPP for SIHR.

	Total number of patients
Short term (<30 days)	
Mortality^a^	1
Early *re-*recurrence <30 days	0
Superficial wound infection	1
Deep wound infection (mesh involved)	0
Haematoma	1
Seroma	1

Long-term (>30 days)	
Late *re*-recurrence	0
Discomfort	2
CPIP	0
Sensory disturbances	0
Sexual discomfort	0

a One patient died 3 weeks postoperatively from sudden cardiac death, not procedure related.

A total of 10 out of 19 patients could be clinically evaluated at a long-term follow-up (mean follow-up 37 months, range 11–95). Reasons for loss to follow-up were death (*n* = 3, of which one <30 days postoperative), inability (*n* = 2), unwillingness (*n* = 2) or inadequate contact information (*n* = 2). None of the patients lost to follow-up had chronic complaints reported in their patient files (*n=*7) or reported CPIP when contacted by phone (*n=*2). From the patients included in the long term follow-up, one patient reported pain when interviewed at the outpatient department. This pain however was previously diagnosed as the result of severe hip arthrosis (confirmed by the patients’ history, physical examination and X-ray). Therefore, no patient with CPIP was diagnosed in this consecutive series. Two patients experienced inguinal ‘discomfort’. No late *re-*recurrences were diagnosed. Additionally, none of the patients experienced a negative impact on their sexual lives from the surgical interventions.

## 4 Discussion

In this small but consecutive case series the feasibility of *re-*TREPP for the repair of a recurrent inguinal hernia after previous TREPP repair was investigated. Although this *re-*TREPP patient group was relatively small and the study design carries clear methodological limitations, results suggest that *re-*entrance of the PPS after a TREPP repair with the TREPP technique seems to be feasible with a low complication rate. In this population none of the patients complained of CPIP related to *re-*TREPP at long-term follow-up and, to date, no *re-*recurrences occurred. The most common cause of recurrence was a malposition of the mesh. The overall peri-operative complication rate was low. There was one conversion from re-TREPP to Lichtenstein due to severely distorted anatomy. No complications occurred during *re-*TREPP.

A previous meta-analysis of randomized trials concluded that the incidence of CPIP is higher after SIHR than after PIHR and that more patients suffer from CPIP after Lichtenstein compared to laparoscopic repair (LoE 1c) [13]. In this study CPIP did not occur, however the sample size is small and therefore prone to bias. The true incidence may be higher, but is hypothetically expected still low because of the ‘stay away from the nerves’-principle of the TREPP technique, in which the inguinal nerves are less at risk compared to an onlay mesh repair such as Lichtenstein’s repair. Although some reports describe that the risk of a recurrence is higher after SIHR compared to PIHR (LoE 2b) [4], no *re-*recurrences occurred. The results after repeated TEP and TAPP techniques were comparable to the presented results (LoE 2b-4), Table 3 [[17], [18], [19], [20], [21], [22]]. Note that the high rate of loss to follow-up may carry a risk of underestimation of the *re-*recurrence rate.

**Table 3:** Previous literature on re-intervention in the PPS.

Author	Year	Type of study	Technique	Number of patients	*Re-*recurrence rate	Level of Evidence
Bisgaard [17]	2008	Prospective cohort	*Re-*TEP and *re-*TAPP	100	7.1% (*n = 1)*	2b
Ertem [18]	2013	Case series	*Re-*TEP and *re-*TAPP	5	0	4
Ferzli [19]	2004	Case series	*Re-*TEP	12	0	4
van den Heuvel [20]	2013	Retrospective cohort	*Re-*TAPP	51	0	2b
Knook [21]	1999	Case series	TAPP after prior endoscopic repair	34	0	4
Leibl [22]	2000	Prospective cohort	*Re-*TAPP	46	0	2b
*This study*	2016	Case series	*Re-*TREPP	19	0	4

Out of the 19 patients with recurrences that were treated with a *re-*TREPP, five occurred within 30 days after the primary TREPP. Although the exact reason for the early recurrence may be difficult to detect, the expert opinion of this group is that they are of technical origin and can be avoided by a more adequate mesh placement in the PPS during PIHR. The mesh should be large enough and requires adequate positioning in the PPS, in order to facilitate proper mesh coverage of all possible hernia orifices [7]. In a narrow space the mesh (with its memory ring) may be comprised, which may therefore cause folding (LoE 5). Therefore accurate development of the delicate PPS is necessary in order to create enough ‘surgical working space’ [7].

Examination of explanted mesh revealed that seven days after implantation the mesh was found firmly integrated to the aponeurosis and partially covered by scar tissue already [23]. The risk of *re-*recurrence may therefore be higher in the first postoperative days, before this process is fully evolved. According to international guidelines, fixation is only recommended for endoscopic repair of large medial hernias to reduce recurrence risk [3]. By Ertem et al (2013), fixation was routinely applied in *re-*TEP and *re-*TAPP to lower the chance of migration [18]. Knook et al (1999) suggested to use a larger mesh, instead of fixation in order to minimise the risk of CPIP [21]. Due to the ‘upstream principle’ the intra-abdominal pressure causes the mesh to be pressed against the abdominal wall [8]. In this series fixation was only performed after clinical judgment during operation in order to create a ‘new’ larger mesh and avoid contact with the tissue (e.g. nerves or nerve brands). To date, this additional value of temporarily fixation in *re-*TREPP surgery remains unclear in this small series and should be interpreted cautiously. The *re-*TREPP procedures were all performed by two dedicated expert hernia surgeons, limiting the external validity and should be cautiously interpreted. Further studies are needed with reliable sample sizes to confirm - or reject - the promising results for *re-*TREPP.

## 5 Conclusion

These first experiences with re-TREPP for secondary inguinal hernia repair are encouraging for the aspects of feasibility and safety, particularly in experienced surgical hands.

## Conflicts of interest

The authors state that they have nothing to disclose.

## Sources of funding

None.

## Ethical approval

None.

According to the Dutch law and in agreement with the rules of the Medical Ethical Board of the Radboud University Medical Centre Nijmegen, no ethical approval was needed for this study (exemption because of study type and concent).

## Consent

Written informed consent was obtained from the patients for publication of this case series and accompanying tables. A copy of the written consent is available for review by the Editor-in-Chief of this journal on request.

## Author contribution

AMP: study concept, design, data collection, data analysis or interpretation, writing the paper.

WJB: data collection, data analysis or interpretation, writing the paper.

WLA: data collection, data analysis or interpretation, writing the paper.

CVL: design, writing the paper, co-supervisor.

GGK: study concept, design, data collection, data analysis or interpretation, writing the paper, supervisor.

## Registration of study

ClinicalTrials.gov, Protocol ID: NCT 03411226.

## Guarantor

Alexandra M. Persoon, MD.

Giel G. Koning, MD, PhD, Supervisor.

## Provenance and peer review

Not commissioned, externally peer-reviewed
